# The Origin and Genetic Variation of Domestic Chickens with Special Reference to Junglefowls *Gallus g. gallus* and *G. varius*

**DOI:** 10.1371/journal.pone.0010639

**Published:** 2010-05-19

**Authors:** Hiromi Sawai, Hie Lim Kim, Kaori Kuno, Sayaka Suzuki, Hideo Gotoh, Masaru Takada, Naoyuki Takahata, Yoko Satta, Fumihito Akishinonomiya

**Affiliations:** 1 Hayama Center for Advanced Studies, The Graduate University for Advanced Studies (Sokendai), Hayama, Kanagawa, Japan; 2 The Graduate University for Advanced Studies (Sokendai), Hayama, Kanagawa, Japan; 3 National Institute of Agrobiological Sciences, Tsukuba, Ibaraki, Japan; 4 The Research Institute of Evolutionary Biology, Setagaya, Tokyo, Japan; Temasek Life Sciences Laboratory, Singapore

## Abstract

It is postulated that chickens (*Gallus gallus domesticus*) became domesticated from wild junglefowls in Southeast Asia nearly 10,000 years ago. Based on 19 individual samples covering various chicken breeds, red junglefowl (*G. g. gallus*), and green junglefowl (*G. varius*), we address the origin of domestic chickens, the relative roles of ancestral polymorphisms and introgression, and the effects of artificial selection on the domestic chicken genome. DNA sequences from 30 introns at 25 nuclear loci are determined for both diploid chromosomes from a majority of samples. The phylogenetic analysis shows that the DNA sequences of chickens, red and green junglefowls formed reciprocally monophyletic clusters. The Markov chain Monte Carlo simulation further reveals that domestic chickens diverged from red junglefowl 58,000±16,000 years ago, well before the archeological dating of domestication, and that their common ancestor in turn diverged from green junglefowl 3.6 million years ago. Several shared haplotypes nonetheless found between green junglefowl and chickens are attributed to recent unidirectional introgression of chickens into green junglefowl. Shared haplotypes are more frequently found between red junglefowl and chickens, which are attributed to both introgression and ancestral polymorphisms. Within each chicken breed, there is an excess of homozygosity, but there is no significant reduction in the nucleotide diversity. Phenotypic modifications of chicken breeds as a result of artificial selection appear to stem from ancestral polymorphisms at a limited number of genetic loci.

## Introduction

When and how domestication proceeded during the history of modern humans are intriguing questions that have attracted much attention from researchers in natural and cultural sciences. Given the progress in genomic sequencing of various animals, the search for target genes of artificial selection during the domestication process has been a focus of recent studies. Because of the relatively short divergence times of domestic animals from their wild ancestors, genomic differences are likely to be subtle. It has therefore been a great challenge to identify genetic changes that resulted from artificial selection during the domestication process. The discovery of genetic differences among domestic breeds should, however, provide some clues. Some recent studies reveal genetic determinants of changes in body size and coat color in dogs [1], [2]. Also, molecular searches for the origins of domestic animals are conducted on other animals such as pigs, sheep, cows, and chickens [3]–[8].

The origins of domestic chickens (*Gallus g. domesticus*) have been debated ever since Darwin [9]. Archeological remains of domestic chickens are found in 16 Neolithic sites along the Yellow River in Northeast China as well as in the Indus Valley. Because some of these remains date back to ∼8,000 years ago [10], domestication must have been undertaken at least since that time. It is suggested that domestic chickens originated from junglefowls in Southeast Asia.

Four species of genus *Gallus* inhabit Southeast Asia: red junglefowl (*G. g. gallus*), La Fayette's junglefowl (*G. lafayettei*), gray junglefowl (*G. sonnerati*), and green junglefowl (*G. varius*). Red junglefowl has a strong sexual dimorphism with males having red fleshy wattles, and it is most widely distributed over the area. La Fayette's junglefowl morphologically resembles red junglefowl, but it inhabits only in Sri Lanka. Gray junglefowl has body plumage on a gray background color and is distributed from southwest to central India. Morphologically distinct green junglefowl is limited to Java and its immediate vicinity, Bali and Lombok. It has been debated whether any single species of the four, especially red junglefowl, predominantly contributed to the genome of domestic chickens (a single-origin hypothesis) or whether multiple species of the four made a substantial genetic contribution to domestic chickens (a multiple-origin hypothesis).

Darwin [9] proposed a single-origin hypothesis based on the observation that only red junglefowl can produce fertile F1 offspring in a cross with chickens. Subsequently, several hybridization experiments were performed to examine the genetic relationship among the four junglefowls and chickens. Danforth [11] reported complete hybrid fertility between red and gray junglefowls. Steiner [12] stated that although F1 females of red and green junglefowls show reduced fertility, they produce F2 hybrids in backcrosses with red junglefowl. In addition, Morejohn [13] found hybridization of gray junglefowl and chickens in the vicinity of villages. Those observations suggested a possible contribution of the four junglefowls to the origin of domestic chickens and favored the multiple-origin hypothesis.

Recently, molecular approaches have been more commonly used to obtain less ambiguous results than classical approaches. Using the D-loop sequences of mitochondrial DNA (mtDNA) in various gallinaceous birds in family Phasianidae, Fumihito et al. [4] concluded that two red junglefowl subspecies, *G. g. gallus* and *G. g. spadiceus*, are the direct ancestors of chickens, but that another subspecies, *G. g. bankiva*, does not contribute. This conclusion was subsequently supported by other studies using microsatellite DNA [14] and a large number of D-loop sequences [15]. Recently, these molecular data also show that Indian red junglefowl (*G. g. murghi*) also contributes to the domestication, as well as *G. g. gallus* and *G. g. spadiceus* [16]. In contrast, the phylogenetic analysis of the entire mtDNA genome and nuclear DNA (nucDNA) regions for four *CR1* (chicken repeat 1) regions and *OTC* (ornithine carbamoyl transferase) revealed evidence for hybridization of gray junglefowl with chickens, red junglefowl, and La Fayette's junglefowl [17]. In particular, the analysis of the entire mtDNA genome showed the presence of identical haplotypes between gray junglefowl and chickens, and the nucDNA analysis of *CR1* and *OTC* loci demonstrated an intermingling clustering pattern among chickens, red and gray junglefowls. These results raised the possibility that junglefowls other than red junglefowl were also involved in the domestication of chickens. In accordance with this possibility, genes responsible for yellow leg skin could be derived from gray junglefowl during the domestication [18].

In this paper, we determine 30 intron DNA sequences at 25 nuclear loci of four domestic chicken breeds consisting of 10 individuals in total. We also determine the orthologous sequences for the sample of four red junglefowls, four green junglefowls and one quail. Based on these sequence data together with the genome database of red junglefowl (build 1.1) and the GenBank database of turkey, we carry out phylogenetic and population genetic analyses. A particular attention is paid to polymorphisms shared among three *Gallus* species that can result from either inheritance of ancestral polymorphisms or introgression.

## Materials and Methods

### Ethics Statement

We adhered to the Guidelines for the Use of Experimental Animals authorized by the Japanese Association for Laboratory Animal Science. All experimental procedures were approved by Institutional Animal Care (National Institute of Agrobiological Sciences) and Use Committee, and all animals were housed and cared for according to guidelines established by the Committee.

### Samples

The samples listed in Table 1 were collected from four chicken breeds (Shamo, sample size *n* = 1; Koshamo, *n* = 4; Ukokkei, *n* = 4; and White Leghorn, *n* = 1), red and green junglefowl species (each with *n* = 4), and quail (*Coturnix japonica*, *n* = 1). Shamo and Koshamo (small Shamo) are breeds of fighting cocks that originated in Thailand and that were imported to Japan by the early Edo period (A.D. 1603–1867). Ukokkei, also known as Silky, uniquely develops one or two extra backward toes. Also given in Table 1 are the locations of our samples and hereafter designated as capitalized abbreviations: SHAMO, KOSHA, UKO, WL, RJF, GJF and QUAIL for samples from each breed and species. CHICKENs mean the whole samples of chicken breeds, namely SHAMO, KOSHA, UKO, and WL, and JFs mean both RJF and GJF.

**Table 1:** Species and breeds used in this study, sampling locations and years, and sample designations.

Species	Latin name	Breed	Sample ID	*n* a	Place of collection (Year of collection)
Domestic chickens	*Gallus g. domesticus*	Shamo	SHAMO	1	Okukuji, Japan (2004)
		White Leghorn	WL	1	NILGS b, Tsukuba, Japan (2004)
		Koshamo	KOSHA151, 152, 153, 154	4	Kagoshima, Tokushima, Japan (1994)
		Ukokkei (Silky)	UKO37, 38, 39, 40	4	Yamagata, Niigata, Okinawa, Japan (1994)
Red junglefowl	*G. g. gallus*		RJF41, 45, 56, 58	4	Palemberg, Indonesia (1994)
Green junglefowl	*G. varius*		GJF301, 302, 303, 304	4	Jakarta, Indonesia (1993)
Quail	*Coturnix japonica*		QUAIL	1	NILGS, Tsukuba, Japan (2004)

a *n* = the number of individuals sampled per species or breed.

b NILGS  =  National Institute of Livestock and Grassland Science.

Whole-blood samples (SHAMO, WL, and QUAIL) or blood blots on filter papers (KOSHA, UKO, RJF, and GJF) were collected from various areas in Japan or Indonesia (Table 1). The blood samples were provided by National Institute of Livestock and Grassland Science (NILGS) in 2004. Animals were maintained on a cycle of 12 h of light and 12 h of darkness. A commercial diet and water were provided. The blood-blotted filter papers of KOSHA and UKO were provided by Dr. Komiyama [19] currently in Tokai University. The filter papers of RJF and GJF were obtained by M. T. All of these were collected in 1993–1994 samples (in 1993, sampled by Dr. Shiraishi in The Research Institute of Evolutionary Biology) and were the same as those used in the previous studies [3], [4]. Sampling in Indonesia was performed with the permission by Indonesian government.

### DNA extraction

Genomic DNA was extracted from whole blood using a Blood & Cell Culture DNA kit (QIAGEN). DNA on the filter paper was in limited quantities, so the following extra procedures were undertaken. To extract genomic DNA, the filter paper was cut into small pieces (3 mm in diameter) with a punch. Several of these pieces were immersed in 10 µl of alkaline lysis solution (400 mM KOH, 100 mM DDT, 10 mM EDTA) and mixed gently. After incubation on ice for 10 min, an equal volume of neutralization solution (400 mM HCl, 600 mM Tris HCl) was added. Genomic DNA was thus dissolved from the filter paper into the solution. An aliquot (1 µl) of this solution was used as a template in the whole-genome amplification, which was performed with the GenomiPhi DNA Amplification kit (GE Healthcare).

### PCR and sequencing

Thirty introns at 24 autosomal and one Z-linked loci (Table 2) were amplified with Ex Taq (TaKaRa) DNA polymerase using primers previously designed for the comparison between chickens and turkeys [20]. The PCR reaction was performed with an initial denaturation at 95°C for 5 min, followed by 30–40 cycles of denaturation at 95°C for 30 sec, annealing at an appropriate temperature for 40 sec, and extension at 72°C for 1 min. Slightly different annealing temperatures were used for each primer set (available upon request). PCR products were purified with a S.N.A.P. Gel purification kit (Invitrogen) or an ExoSAP-IT kit (Amersham Biosciences). Purified products were directly sequenced or cloned (heterozygous samples) using a TOPO TA cloning kit or a TOPO XL PCR cloning kit (Invitrogen). To obtain reliable sequence data, 8–12 clones were sequenced for each PCR product except for those from SHAMO, WL, and QUAIL. For these three samples, the number of sequenced clones was limited to less than six for technical reasons, and a single sequence was used as a representative of each sample. The BigDye Terminator Cycle Sequencing kit (Applied Biosystems) was used with the corresponding PCR primers. Sequencing was carried out on ABI377 and ABI3100 sequencers (Applied Biosystems). Sequences were read at least twice in both directions. The nucleotide sequences obtained were deposited into DDBJ (accession numbers AB495408–496406).

**Table 2:** Chromosomal locations and nucleotide lengths of the introns sequenced in this study.

Intron	Locus (abbreviation)	Length (bp)	Chromosome	GeneID a
1	Adenylate kinase (*AK1int3*)	376	17	D00251
2	Annexin V, intron 5 (*ANXA5int5*)	821	4	428767
3	Annexin V, intron 7 (*ANXA5int7*)	416	4	428767
4	Crystalline, beta A1 (*CRYBA1int2*)	801	19	396499
5	Hemoglobin, alpha D (*HBADint2*)	315	14	416651
6	Creatine kinase, brain (*CKBint3*)	529	5	396248
7	Actin, beta, intron 2 (*ACTBint2*)	485	2	X00182
8	Actin, beta, intron 3 (*ACTBint3*)	312	2	X00182
9	Transcriptional repressor-delta EF1 (*DEF1int3*)	665	2	396029
10	Fatty acid synthase (*FASNint41*)	1335	18	396061
11	Growth hormone 1 (*GH1int2*)	449	27	378781
12	Glyceraldehyde-3-phosphate dehydrogenase (*GAPDHint2*)	286	1	374193
13	Heat shock 108-kDa protein 1 (*HSP108int2*)	698	1	374163
14	Interleukin 8 (*IL8int3*)	571	4	396495
15	Ribosomal protein-coding gene L37A (*RPL37Aint3*)	1121	7	769981
16	Ribosomal protein-coding gene L5 (*RPL5int3*)	613	8	395269
17	Ribosomal protein-coding gene L7A, intron 3 (*RPL7Aint3*)	358	17	417158
18	Ribosomal protein-coding gene L7A, intron 4 (*RPL7Aint4*)	351	17	417158
19	Luteinizing hormone/choriogonadotropin receptor (*LHCGRint7*)	513	3	395776
20	Myosin light chain (*MLCint4*)	477	7	396470
21	Nicotinic acetylcholine receptor, gamma-subunit (*ACGRint7*)	708	9	429151
22	Opsin intron 1 (*OPN1LWint1*)	528	19	396377
23	Opsin intron 3 (*OPN1LWint3*)	227	19	396377
24	Opsin intron 4 (*OPN1LWint4*)	381	19	396377
25	Rhodopsin visual pigment intron 4 (*RHOint4*)	879	12	396486
26	Ribosomal protein-encoding gene L30 (*RPL30int2*)	1006	2	425416
27	Transforming growth factor-beta 2 (*TGFB2int7*)	561	3	421352
28	Vimentin (*VIMint5*)	625	2	420519
29	Spindlin on Z (*SPINZint3*)	957	Z	395344
30	Clathrin heavy chain (*CLTCint7*)	676	19	395272

a The genome data is build 2.1. For three cases (intron 1, 7, and 8) of which Gene ID is not available, accession numbers are indicated.

### Phylogenetic analysis

Orthologous intron sequences were retrieved from the red junglefowl genome database (build 1.1) and those of turkey (*Meleagris gallopavo*) from GenBank (accession numbers: AY139863, AY139865, AY142943, AY142944, AY144673–AY144682, AY194143, AY298973–AY298989, AY3807788, AY380789). As before, these sequences are designated as RedDB and TURKEY. All the sequences were aligned using CLUSTAL W [21] and then adjusted manually whenever necessary. On the other hand, for the mtDNA D-loop, WL (AP003317), UKO (AB113070, AB086102), KOSHA (AB098676, AB098672, AB098671), RJF (NC_007236, AB007720), GJF (NC_007238, EU329408), TURKEY (NC_010195, AF532414), and QUAIL (NC_003408, NC_004575, X57245) sequences were used.

**Figure 1:**
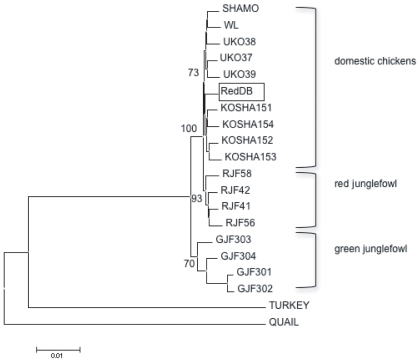
The *average-difference* tree based on 1,000 concatenated sequences of randomly selected diploid sequences. The proportion supporting a cluster is shown at each node as the realization of that cluster in the 1,000 *individual* trees. The TURKEY and QUAIL sequences are used as outgroups. The boxed RedDB indicates that the sequences were taken from the database of red junglefowl. Significant contributions of the domestic chicken genome to this database sequence are evident. The scale shown below the figure is a branch length corresponding to a per-site number of substitutions of 0.01 (1%). Abbreviations for samples are as follows. SHAMO: Shamo, WL: White leghorn, UKO: ukokkei, KOSHA: Koshamo, RJF: red junglefowl, GJF: green junglefowl.

It is to be noted that only one sequence at each intron is available for SHAMO, WL, RedDB, QUAIL, and TURKEY, and thus the sequences from various introns of these individuals were uniquely concatenated. In contrast, the diploid sequences were determined at the 29 autosomal introns for most of KOSHA, UKO, RJF, and GJF (Table S1). Because we do not have any phase information among different introns, one of the diploid sequences at each intron was randomly selected and concatenated to represent an individual sample (a randomly concatenated sequence). For a set of 21 randomly concatenated sequences (four KOSHAs, four UKOs, four RJFs, four GJFs, SHAMO, WL, RedDB, QUAIL, and TURKEY), an *individual* tree was constructed based on the *p*-distances or the per-site nucleotide differences in a pair of individuals and the neighbor-joining (NJ) method [22]. The procedure of producing 21 randomly concatenated sequences was repeated to obtain 1,000 *individual* trees (Figure S1). As a summary of phylogenetic information, the individual *p*-distances were averaged over the 1,000 repeats (*p*_a_ distances) and then used to construct the *average-difference* tree (Figure 1). Assignment of a cluster in the *average-difference* tree was measured as the proportion of its occurrence among the 1,000 *individual* trees. The value reflects the robustness of clustering in 1,000 *individual* trees.

### Demographic analyses

Genetic variation within and between species was measured by the nucleotide diversity (π) and the nucleotide divergence (*d*), respectively. The subscripts of C, R, and G for π stand for domestic chickens, red and green junglefowls, respectively, and those for *d* stand for two species compared. To estimate the nucleotide divergences of mtDNAs (*d*_mt_) and nucDNAs (*d*_nuc_) as well as the nucleotide diversity (π_mt_ and π_nuc_), Jukes and Cantor [23] or Kimura's two-parameter method [24] were used for multiple hits correction.

The “Structure” program by Pritchard et al. [25] was applied to examine the genetic ancestry of 16 samples of KOSHA, UKO, RJF, and GJF with observed genotypes at each intron. In the application, 10,000 iterations were performed for each of the burn-in and thereafter.

The Markov chain Monte Carlo (MCMC) method [26] and the two-species maximum-likelihood (TSL) method [27], [28] were applied to estimate the effective size of the ancestral and extant populations (*N*_e_) and the species divergence time (*t*_s_). In both methods, the estimates were scaled by the per-year nucleotide substitution rate (μ): θ = 4 *N*_e_µ*g* and τ = *t*_s_µ, where *g* is the generation time in units of years. In the MCMC method, all the diploid sequences at each intron were used simultaneously. This method requires a gamma distribution to specify the prior distributions of θ and τ, which were calculated from the actual sequence data. We used two different sets of parameters (Table S2) to confirm the robustness of our estimates. For each parameter set, six runs were applied with a different set of random numbers. For each run, the number of iterations for the burn-in was 10,000, and the number of iterations after the burn-in was 200,000, with a thinning of two. Thus, coalescence-based genealogies were generated 100,000 times, from which the mean, standard errors, median, and 95% confidence limits of estimates were calculated.

The TSL method restricts the number of sequences at a locus to one for each species under study. For this restriction, a sequence was randomly selected from multiple sequences at an intron in each species. This random sampling process was repeated 1,000 times for each pair of species, and 1,000 maximum-likelihood (ML) estimates of θ and τ were obtained, from which the mean, standard error, and median were calculated. Our program for the ML estimation of θ and τ is written in Mathematica (version 4.0, Wolfram Research) and is available upon request.

## Results

### Phylogenetic analysis of concatenated diploid sequences

We examined one sample of each of SHAMO, WL and RedDB and four samples of each of UKO, KOSHA, RJF and GJF (Table 1). Unfortunately, not all the 30 intron sequences (Table 2) could be obtained from some individuals (see Table S1 for details). Most unsuccessful was UKO40 for which we could not determine the nucleotide sequences at nine introns and therefore excluded from the subsequent analyses. Thus, the total number of *Gallus* samples in the following analysis was 18.

Using the TURKEY and QUAIL as outgroup sequences, we compared 1,000 *individual* trees (Figure S1). In most of the trees, RJFs formed a single cluster, whereas RedDB was placed within a cluster of chicken breeds. Several substitutions were specific to a majority of RJFs. While these substitutions were responsible for their tight clustering, they were not shared with RedDB. As noted in the database, RedDB might not be pure red junglefowl, but a hybrid with a chicken breed (possibly White Leghorn). In what follows, we excluded RedDB.

Importantly, species-specific clustering was not seen for GJFs for many individual trees. Two samples of GJF303/304 (Table 1) were often assigned to a cluster of chicken breeds (e.g., individual trees 8, 9, and 1 in Figure S1) so that the introgression occurred from chickens to green junglefowl. The average *p*_a_ distance (Table S3) of GJF303/304 from CHICKENs (UKO, KOSHA, WL and SHAMO) was 1.2±0.1%, significantly smaller than 1.7±0.1% in the case of GJF301/302 from CHICKENs. Although the *average-difference* tree (Figure 1) showed that the GJFs were reciprocally monophyletic to the RJFs and the CHICKENs, the branch lengths leading to GJF303/304 were apparently shorter than those leading to GJF301/302. This monophyletic relationships were supported by individual trees as well, but the proportion was 93%, 73% and 70% for the monophyletic cluster of RJFs, CHICKENs and GJFs, respectively (Figure 1).

Within the CHICKEN cluster, four KOSHAs formed a separate clade. Although the proportion of individual trees supporting this clade was low (30%), none of KOSHAs shared any haplotypes with any other CHICKENs at introns 11, 20, 21, and 25 (Table S1). This observation indicated that genetic differentiation in some genomic regions had indeed taken place among different chicken breeds.

### NucDNAs vs. mtDNAs

To estimate the substitution rate of nuclear DNA in domestic chickens and junglefowls, we tested whether *d*_nuc_ accumulated proportionally to elapsed time (see [29] for review). Actually, in the lack of solid information on speciation time, we tested if *d*_nuc_ is proportional to *d*_mt_.

In doing such a test, we first noted that the Phasianidae mtDNA D-loop region was subjected to different degrees of sequence conservation. The region was divided into three domains, each of which contained a unique conserved nucleotide sequences, and because of such conservation, the D-loop region as a whole evolved relatively slowly [30] (see also [3], [31] for other galliforms and anseriforms). However, the sequence alignment clearly showed hypervariability in a certain region (data not shown). It was difficult to align the sequences from position 172 to 382 between chickens and quails/turkeys [30] and even between chickens and green junglefowl. For this reason, we omitted this 210 bp region from the analysis.

The sequence divergence between two related species reflected the nucleotide substitutions that accumulated after the speciation in addition to ancestral polymorphism [27], [32]. Even when the nucleotide substitutions accumulate in a steady fashion, the proportionality between nucDNA and mtDNA divergences does not hold true owing to ancestral polymorphism. A frequently used method for estimating the nucleotide divergences after speciation assumes that the extent of ancestral polymorphism is equal to that of extant populations [32], [33] and subtracts this from the sequence divergence between species. In the present study, however, this assumption was not warranted because domestication had likely influenced the demography of domestic chickens. Instead, we directly compared *d*_nuc_ and *d*_mt_ in distantly related pairs in Phasianidae where the effect of ancestral polymorphism on the divergence could be small enough to be ignored without serious errors.

**Figure 2:**
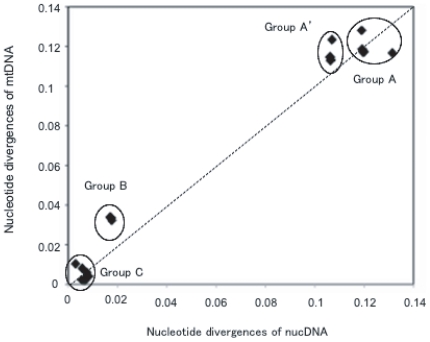
Nucleotide divergence among mtDNA D-loop (*y* axis) and nuclear intron sequences (*x* axis). The multiple-hit substitutions were corrected by the method of Jukes and Cantor [23] and Kimura [24]. Group A and A′ comprise comparisons of QUAIL and TURKEY, respectively vs. *Gallus* samples. Group B comprises comparisons of GJFs vs. RJFs and chickens. Group C represents inter- and intra-group comparisons of RJFs and chickens. The dotted line indicates *d*_mt_ = *d*_nuc_. Each individual data point represents the comparison of a single species pair. Abbreviations for samples are the same as in Figure 1.

Figure 2 plotted *d*_nuc_ and *d*_mt_ for pairs of species, revealing the presence of four distinct groups (Group A, A′, B, and C). Groups A and A′ compared most distantly related species pairs between CHICKENs or JFs (RJFs and GJFs) and QUAIL or TURKEY, respectively. Group B compared GJFs with CHICKENs or RJFs, whereas Group C compared RJFs and CHICKENs as well as samples within CHICKENs or JFs. Thus, Group C included intra-specific comparisons.

In both Groups A′ and B, *d*_mt_ was slightly larger than *d*_nuc_. A greater departure from *d*_mt_ = *d*_nuc_ occurred in Group B than in Group A′, suggesting that even the conserved D-loop region underwent multiple-hit substitutions in more distantly related comparisons. Indeed, in the comparison of Group A′, 20 out of 261 variable sites possessed either three different kinds of nucleotides or phylogenetically incompatible substitutions at each site. This trend became more obvious in the most distantly related comparisons of Group A. A point was that *d*_nuc_ in Group B and C increased almost linearly as *d*_mt_ increased. We took this as evidence for the presence of molecular clock, or more precisely, if a mitochondrial clock exists in Phasianidae, so does a nuclear clock.

### Shared and species-specific haplotypes

The number of haplotypes per intron per breed or species ranged from one to 11. Although this wide range certainly reflected the differences in sample size, the observed number of haplotypes fitted well with the expected number based on the sampling theory [34] (Table 3).

**Table 3:** Number of haplotypes (*H*) and the percent nucleotide diversity (π) in the 30 introns.

Introns	Chickens					RJF					GJF				
	*n* a	p	O(*F*)^b^	*H*	E(*H*)^c^	*n*	p	O(*F*)	*H*	E(*H*)	*n*	p	O(*F*)	*H*	E(*H*)
1	18	0.61	6	7	5.5	8	0.82	1	4	4.5	8	0.37	1	5	3.2
2	17	0.67	2	8	7.9	8	1.05	2	3	6.4	8	0.73	2	3	5.7
3	18	0.66	2	8	6.1	8	0.66	2	3	4.4	8	0.95	0	5	5
4	10	0.30	3	3	4.5	6	0.26	0	4	3.5	–d	–	–	–	–
5	18	0.47	7	2	3.9	8	0	4	1	1	8	0.33	2	3	2.6
6	18	0.25	3	6	4.1	8	0.23	1	4	3.1	8	0.13	1	3	2.4
7	18	0.33	5	5	4.6	8	0.27	0	5	3.2	8	0.56	2	5	4.4
8	16	1.14	6	6	6.5	8	0.85	0	5	4.3	8	0.90	2	4	4.4
9	16	0.71	4	6	7.4	8	0.82	0	4	5.5	8	0.79	1	4	5.5
10	18	1.26	4	9	12.7	8	1.06	1	5	7.1	8	1.37	0	7	7.4
11	18	0.65	5	10	6.3	8	0.69	1	5	4.6	8	0.40	2	3	3.6
12	18	0.50	5	6	4.2	8	0.83	2	3	4.1	8	1.65	2	4	5.3
13	16	1.26	4	7	9.8	8	1.63	2	5	6.8	8	1.79	0	6	7
14	18	0.72	3	8	7.5	8	0.43	2	3	4.2	8	1.17	2	4	6
15	16	0.42	5	6	6.7	8	0.65	3	3	5.7	8	1.93	2	4	7.3
16	18	0.58	4	6	7	8	0.40	2	3	4.2	8	0.67	2	5	5.1
17	18	0.49	5	8	4.8	8	0.65	2	4	4.1	8	1.93	0	7	6.1
18	18	0.67	7	8	5.6	8	0.28	3	3	2.7	–	–	–	–	–
19	18	0.82	5	5	7.5	8	0.56	1	4	4.4	8	1.00	1	5	5.5
20	18	0.85	6	8	7	8	0.31	2	4	3.4	8	1.23	1	6	5.7
21	16	0.40	2	9	5.9	8	0.24	4	2	3.6	8	0.71	1	5	5.5
22	16	0.72	7	6	6.8	8	0.89	1	3	5.3	8	1.17	2	3	5.8
23	18	0.86	5	6	5.1	8	0.87	0	3	3.8	8	0.57	2	3	3.1
24	18	0.49	6	6	5	8	1.90	3	4	6.1	8	1.90	1	4	6.1
25	18	0.57	4	9	8.2	8	0.48	1	6	5.2	8	0.60	2	6	5.6
26	16	1.09	2	10	9.5	8	1.08	1	4	6.3	8	1.77	0	8	7.1
27	18	0.12	8	4	2.9	8	0.33	2	3	3.7	8	0.71	0	4	5.1
28	18	1.22	4	6	9.8	8	0.27	2	2	3.5	8	0.63	0	5	5
29	13	0.08	–	3	2.7	7	0	0	1	1	8	0.15	–	4	2.8
30	18	0.47	7	5	6.6	8	0.36	1	5	4.2	8	0.86	2	4	5.7

a *n* =  the number of sampled chromosomes.

b O(*F*) =  the observed number of homozygotes.

c E(*H*) =  the expected number of haplotypes or alleles based on π*L* and Ewens's sampling theory [34], where *L* is the length of an intron, which is given in Table 2.

d A dash indicates that the sequences were not obtained in this study.

**Figure 3:**
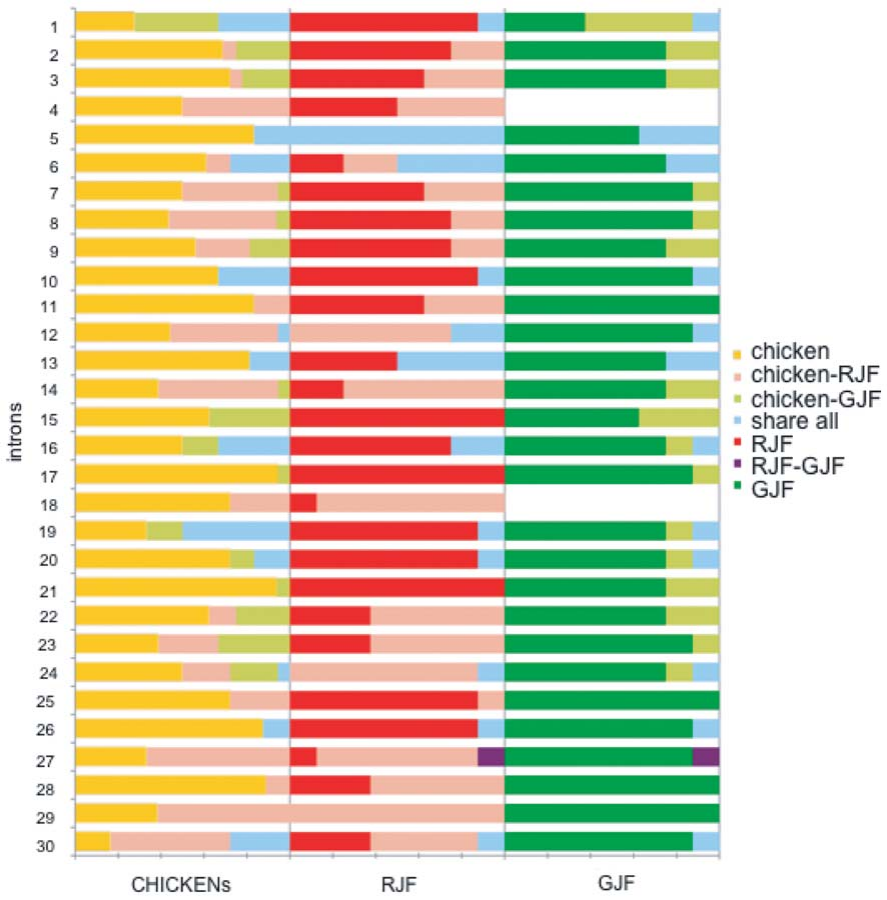
The proportion of shared haplotypes at 30 introns. The 30 intron numbers in Table 2 are shown on the left. Different colors indicate different patterns of shared haplotypes as specified by the key on the right margin. CHICKENs in this Figure include only UKO and KOSHA samples. Abbreviations for samples are the same as in Figure 1.

To quantify the extent of genetic information shared between CHICKENs and JFs, we examined the proportion of shared haplotypes at each intron (Figure 3). The average proportion of shared haplotypes per intron was about 21% between CHICKENs and RJFs, and it was about 11% between CHICKENs and GJFs. Thirteen haplotypes at 12 loci were shared among the three species groups, 24 at 19 loci shared between RJFs and CHICKENs, and 18 at 16 loci shared between GJFs and CHICKENs. In the case of GJFs, all of the shared haplotypes were represented in either GJF303 or GJF304, or both. In one case, a haplotype at intron 1 (*AKI*) shared with CHICKENs was found not only in both GJF303 and 304 but also in GJF301. In contrast to a relatively high proportion of haplotypes shared between CHICKENs and JFs, haplotypes shared between RJFs and GJFs were so rare that there was only one at intron 27 (*TGFB2*).

Of course, there existed species- or breed-specific haplotypes as well. The proportion per intron ranged from 20% to 90% in CHICKENs, but for a particular breed at least one haplotype at each intron was shared with another breed or JFs. In contrast, there were species-specific haplotypes only at three introns (*RPL37A*, *RPL7Aint3*, *ACGR*) in RJFs and four introns (*GH1*, *RHOint4*, *VIM*, and *SPINZ*) in GJFs. As expected, the proportion of species-specific haplotypes was higher in GJFs than in RJFs.

### Genetic variation within and between species

We estimated the nucleotide diversity (π) and divergence (*d*) at 30 introns within and between domestic chickens and junglefowls (Table 3, Table S4). The average π value was π_C_ = 0.65±0.32% for CHICKENs and π_R_ = 0.58±0.37% for RJFs. On the other hand, π_G_ = 0.97±0.56% for GJFs and six introns (*GAPD*, *HSP108*, *RPL37A*, *RPL7Aint3*, *OPSINint4*, *RPL30*) individually exhibited high π values (>1.7%). However, if GJF303/304 were excluded, the π values at *GAPD*, *RPL37A*, *OPSINint4*, and *RPL30* decreased substantially (Figure S2). The π_G_ values at *HSP108* and *RPL7Aint3* introns were 1.8±1.1% and 1.9±1.4%, but they remained high at 1.7±1.0% and 1.3±0.9% even if GJF303/304 were removed. The cause of this large π_G_ was probably due to a presence of haplotypes which were more closely related to CHICKENs or RJFs than to other haplotypes in GJFs.

### Population structure

**Figure 4:**
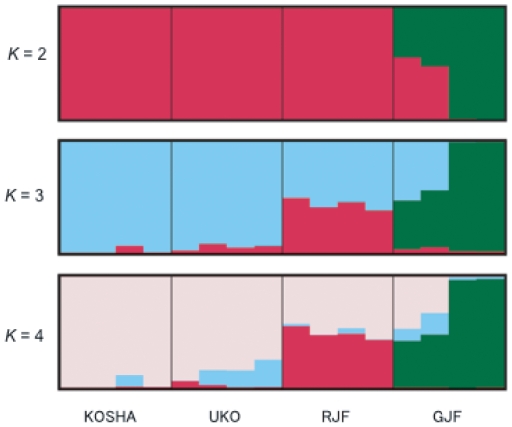
‘Structure’ graph for individual proportions of shared ancestry. Ancestry was estimated from diploid intron sequences in the samples from four KOSHAs, four UKOs, four RJFs, and four GJFs. Predefined populations are shown on the abscissa. Different colors within each block indicate different ancestries. Abbreviations for samples are the same as in Figure 1

To obtain an overview of the genetic components of CHICKENs, RJFs, and GJFs, we used the program Structure [25]. We tested several different models, but the results did not differ greatly. Figure 4 shows the result of the ‘admixture model’ with ‘allele frequency correlations.’ The most likely value of *K* was estimated as 2, suggesting that genetic components in these samples were likely to be classified into two groups. The first component was found mainly in RJFs and CHICKENs, and partially in GJFs, whereas the second component was exclusively found in GJFs. In this regard, RJFs and CHICKENs did not differ much genetically. In contrast, for *K* = 3 or 4, RJFs and CHICKENs became distinguishable. On one hand, approximately a half of the RJF genome was characteristic of the species, and a substantial portion of CHICKENs penetrated into RJFs and GJFs. On the other hand, GJFs and RJFs shared few genetic components, which was consistent with the low proportion of shared haplotypes (Figure 3). It appeared that unlike chickens, red and green junglefowls had been strongly isolated from each other since their speciation.

### MCMC and TSL analyses

We estimated the species divergence time (*t*_s_) and the effective size (*N*_e_) of the ancestral and extant populations from the present data. There are several such estimation methods including the TSL [27], [28], the tree mismatch [32], and the MCMC [26] methods. Because multiple sequences were available from each species, we preferred the MCMC method that does not give any limitation to the number of sequences from a species. Nevertheless, we were interested in comparing the TSL estimates with the MCMC estimates.

**Figure 5:**
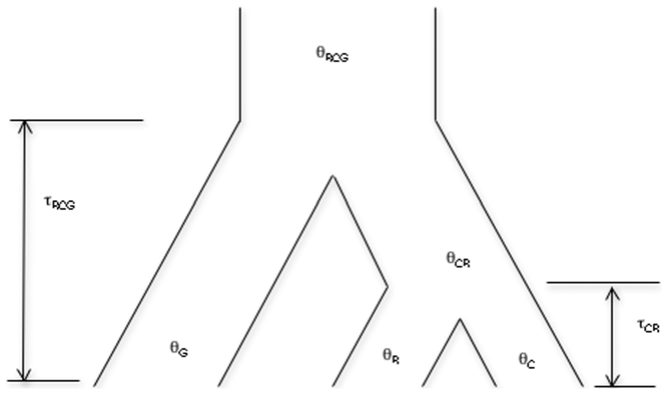
The three species and demographic parameters as estimated by MCMC and TSL methods. With the TSL method, only pairs of species are taken into account at once, such that one of the three consecutive subscripts of a demographic parameter is shown in parentheses in the text. The symbols of θ and τ stand for the nucleotide divergence due to the extent of polymorphism and the species divergence, respectively. The subscript G, R, C means the three species, green junglefowl, red junglefowl and domestic chickens, respectively.

In the case of three species (Figure 5), θ = 4 *N*_e_µ*g* and τ = *t*_s_µ were concerned with two species-divergence times (τ_CR_ and τ_RCG_) and five effective sizes of ancestral and extant populations (θ_C_, θ_R_, θ_G_, θ_CR_, and θ_RCG_). We conducted several independent runs of the MCMC method with different parameter sets in the prior distribution. The estimates were not much different for different parameter sets (data not shown).

We compared the MCMC results with and without GJF303/304. In their exclusion, both θ_G_ and θ_RCG_ decreased substantially and the decrease of θ_RCG_ was compensated by an increase in τ_RCG_. We excluded GJF303/304 for further analyses. In the TSL method, we used 1,000 sets of randomly selected diploid sequences at each intron for each species (Table 4). For distantly related species, the MCMC method mostly resulted in *d*<θ+2τ, but the TSL method almost always resulted in *d* = θ+2τ [27], [28]. For closely related species, the two methods also resulted in different estimates of θ and τ, although θ+2τ did not differ much from the average nucleotide divergences.

**Table 4:** Comparison of means ± standard deviations between MCMC (Markov chain and Monte Carlo) and TSL (two species likelihood) estimates (%) based on 26 intron sequences with and without GJF303 and 304.

Estimates^a^	MCMC^b^ [95% confidence limits]	TSL [median]^c^	
With GJF303 and 304			
θ_C_	0.24±0.06 [0.13, 0.37]		–
θ_R_	0.10±0.03 [0.05, 0.16]		–
θ_G_	0.58±0.08 [0.43, 0.75]		–
θ_CR_	1.60±0.14 [1.34, 1.90]		0.58±0.16 [0.58]
θ_RCG_	1.94±0.30 [1.43, 2.58]		θ_CG_ = 1.10±0.25 [1.07],θ_RG_ = 1.06±0.22 [1.04]
τ_CR_	0.01±0.003 [0.007, 0.02]		0.13±0.08 [0.12]
τ_RCG_	0.12±0.01 [0.10, 0.14]		τ_CG_ = 0.31±0.15 [0.32],τ_RG_ = 0.31±0.13 [0.33]
Without GJF303 and 304			
θ_C_	0.26±0.06 [0.15, 0.39]		–
θ_R_	0.11±0.03 [0.06, 0.18]		–
θ_G_	0.34±0.06 [0.25, 0.46]		–
θ_CR_	0.71±0.14 [0.47, 1.00]		0.57±0.16 [0.58]
θ_RCG_	1.61±0.15 [1.34, 1.92]		θ_CG_ = 0.80±0.10 [0.80],θ_RG_ = 0.86±0.08 [0.86]
τ_CR_	0.01±0.003 [0.008, 0.02]		0.13±0.08 [0.12]
τ_RCG_	0.63±0.06 [0.52, 0.74]		τ_CG_ = 0.59±0.03 [0.58],τ_RG_ = 0.55±0.02 [0.55]

a The symbols of θ and τ stand for the nucleotide divergence due to the extent of polymorphism and the species divergence, respectively. θ = 4 *N*_e_µ*g* and τ = *t*_s_µ, where *g* is the generation time in units of years, *N*_e_ is the effective population size, *t*_s_ is the species divergence time and µ is the per-year nucleotide substitution rate.

b Scale and shape parameters for gamma distributions of prior MCMC analyses are given in Table S2, and the result for set 1 is shown here.

c Two different pairs of species were used for the TSL method. The estimates for the pair of chickens and green junglefowl are shown as θ_CG_ and τ_CG_, and those for the pair of red and green junglefowls are indicated as θ_RG_ and τ_RG_.

## Disucussion

### Inferring a species tree from gene trees

There are several methods for inferring a species tree from multiple gene trees. Generally, these methods can be classified into one of two approaches: the combined or separate approach. In the combined approach, sequences from multiple loci are concatenated, and the resulting data set is analyzed using phylogenetic methods. In the separate approach, the sequence data from each locus is first analyzed individually, and then a reconciliation of different gene trees is obtained.

In this study, we used a slightly modified version of the combined approach. Because of diploid sequences available at 26 loci and because of numerous phase combinations per individual, a consensus tree was constructed based on the average distances of 1,000 sets of random sampling of concatenated sequences (the *average-difference* tree; Figure 1). Basically, each topology of 1,000 *individual trees* based on individual sampling is similar to that of the *average*-*difference* tree. In contrast, when a tree at each intron is constructed separately, the resulting trees shows that the individual samples are intermingled with each other and do not have a sufficient power to distinguish among CHICKENs, RJFs, and GJFs. In the lack of phase information, combining data at different loci is advantageous to minimize noise [35], [36]. The *average-difference* tree clearly shows that there is a monophyletic relationship among chickens, red and green junglefowls and that chickens are more closely related to red junglefowl than green junglefowl.

### Estimation of species divergence times from *d*_mt_ and *d*_nuc_

The relationship between *d*_mt_ and *d*_nuc_ shows a rough linearity, although there is some indication of saturations in *d*_mt_ when distantly related species are compared (Groups A and A′ in Figure 2). To avoid the saturation effect as much as possible, we used the divergences in Group B with *d*_nuc_ = 1.9±0.9% and *d*_mt_ = 3.3±0.1% (Figure 2). The ratio of *d*_mt_ to *d*_nuc_ is 1.7, indicating that the conserved mtDNA D-loop region of *Gallus* evolves only 1.7 times faster than nucDNA. This is in sharp contrast to mammals where the D-loop region may have evolved approximately 100 times faster than nucDNA [37]. Thus, even for such deep phylogenies as those in family Phasianidae, mtDNA together with nucDNA can give reliable evolutionary signals.

We used the divergence time between quails and chickens to calibrate the absolute nucDNA substitution rate. Three independent but similar estimates of the divergence time were available: The fossil record dates the species divergence to 32–38 million years (myr) ago [38], mtDNA sequences to 33–36 myr ago [39], and micro-complement fixation analyses to 38 myr ago [40]. We assume the divergence time to be 35 myr ago. Using the average *d*_nuc_ = 12±0.5% in the comparison of chickens with quails and ignoring ancestral polymorphisms, we estimate the rate as 1.7±0.07×10^−9^ per site per year. This rate is almost the same as that in the previous report [20] and slightly greater than the rate in primates [28]. Likewise, the mtDNA substitution rate is simply 1.7 times higher and estimated as 2.9×10^−9^ per site per year.

The above calibrated rate and *d*_nuc_ = 11±0.03% between turkeys and junglefowls lead to their divergence time of 31±0.7 myr. Likewise, *d*_nuc_ = 1.9±0.9% between green junglefowl and chickens indicates the divergence time of 5.5±2.7 myr, which is more recent than the previous estimates [39] (see also [41], [42]). In either case, the actual divergence time may be even shorter than what the *d* value indicates, because *d* for closely related species might be substantially increased by ancestral polymorphism.

### Demographic history of green junglefowl and introgression of the chicken genome

The MCMC and TSL methods attempt to remove the effect of ancestral polymorphisms on estimates of the species divergence time. The MCMC estimate of τ_RCG_ is 0.63±0.06% (Table 4) and this is indeed smaller than *d*_nuc_/2 = 0.95%. The net species divergence time (*t*_RCG_) of green junglefowl becomes 3.6 myr (95% confidence interval is 3.0–4.3 myr). Similarly, the TSL estimate of τ_ CG_ or τ_RG_ is 0.59±0.03% or 0.55±0.02%, which indicates that *t*_ CG_ = 3.4±0.2 myr or *t*_ RG_ = 3.2±0.1 myr. Both the MCMC and TSL estimates of *t*_RCG_ are thus in agreement with each other. These estimates are smaller than not only 4–5 myr based on the fossil record (see [37] and references therein), but also the *d*_nuc_-based estimate of 5.5 myr. On the other hand, they are consistent with a volcanic origin of Java in the Plio-Pleistocene era (3–4 myr). Because green junglefowl is limited to Java and its immediate vicinity, the speciation may have coincided with this geological event [41].

The presence of haplotypes shared with chickens can be explained by ancestral polymorphisms and/or introgression. To distinguish these two causes, we calculate the probability (*P*_C_) that two shared haplotypes are identical by descent for a given time duration. Using μ = 1.7×10^−9^ per site per year and the MCMC estimate of *t*_RCG_ of 3.6 myr, *P*_C_ becomes <0.005 if the sequence length is >250 bp. This indicates that a pair of orthologous sequences can be identical with the probability of less than 0.005. Among the 26 intron sequences analyzed, 25 introns are >250 bp long, and 22 or 17 haplotypes at the 25 introns are identical between chickens and GJF303 or 304. We therefore conclude that these identical sequences found between chickens and green junglefowl are caused by recent introgression of the chicken genome into green junglefowl.

The multiple-origin hypothesis of domestic chickens postulates different junglefowl species that were involved in the origin of domestic chickens. Although red and green junglefowls can still hybridize with chickens and the genome of one species is actually transferred over the species boundary, these observations are not direct evidence for the multiple-origin hypothesis of domestic chickens. Rather, green junglefowl has been genetically distinct from chickens since green junglefowl speciated around the time of the uplift of the Java islands.

The possibility that other species such as La Fayette's and gray junglefowls were involved in the domestication remains to be elucidated. Unfortunately, our samples include neither of these junglefowls nor any other subspecies of red junglefowl. We do, however, hope that we will be able to depict a clear picture once such samples are used in the present framework.

### Ancient divergence and a large ancestral population of red junglefowl

At 27 out of 30 introns in red junglefowl, shared haplotypes with chickens are observed. The proportion of shared haplotypes at each intron is much higher in red junglefowl than in green junglefowl. Importantly, these shared haplotypes are evenly distributed over all samples of red junglefowl, whereas this is not the case for those of green junglefowl.

According to archeological findings, the divergence time of domestic chickens from junglefowls is estimated to be on the order of 10,000 years. The MCMC method reveals, however, that the extent of nucleotide divergence after the split of red junglefowl from the chicken ancestor is as small as τ_CR_ = 0.01% (Table 4). With the calibrated μ, the divergence time *t*_CR_ becomes 58,000±16,000 years ago. This dating is nearly six times older than what the archeological remains suggest. Because the MCMC model assumes the complete separation of species, it may still underestimate *t*_CR_ if subsequent introgression occurred. The relatively small 95% confidence limit of the MCMC estimate (Table 4) does not reconcile the discrepancy. In any case, more intron sequences from appropriate samples from red junglefowl will be needed to prove or disprove the rather old species divergence of red junglefowl.

During this period of divergence time, the expected number of substitutions is less than one even in the longest intron in our sample. It is thus most likely that shared haplotypes are identical by descent. Nonetheless we cannot exclude the possibility of introgression. Given that introgression takes place between green junglefowl and chickens, it is even more likely to occur between a more closely related pair of species, red junglefowl and chickens.

### The genetic variation within chicken breeds

The MCMC estimate of θ_C_ = 0.26% for the entire CHICKENs is twice as large as θ_R_ = 0.11% for RJFs (Table 4). An important implication of θ_C_ is that distinct ancestral lineages of red junglefowl could have been used for domestication. Again, with the calibrated μ and the generation time (*g*) of one year, the effective size of domestic chicken population becomes as large as 4×10^5^. This estimate is 2.5 times larger than the estimate of 1.6×10^5^ for the extant red junglefowl population, but it is slightly smaller than the estimate of 5×10^5^ for the extant green junglefowl population. On the other hand, the effective size of the ancestral population of red junglefowl and chickens is estimated as 10^6^ from θ_CR_ = 0.71% (with a 95% confidence interval of 0.7–1.4×10^6^) and the ancestral size of three species is estimated as 2.3×10^6^. Compared with these values, the effective population size has been reduced to less than half in all the three extant species.

The genetic variation maintained within chicken breeds is also of particular interest. Koshamo and Ukokkei clearly show an excess of homozygotes as compared with the Hardy-Weinberg equilibrium (Table S5). Out of eight samples, the number of expected and observed homozygotes averaged over 29 autosomal introns is 2.2 and 4.7, respectively. This excess most likely reflects intensive inbreeding within each breed. However, the π value within Koshamo (0.52±0.36%) and Ukokkei (0.63±0.35%) is actually similar to the value for chickens as a whole, 0.65±0.32%. These values are also similar to the π values estimated for other breeds using SNP data across the entire genome [43]: 0.43% for Broiler, 0.37% for Layer, and 0.55% for Ukokkei. Clearly, each individual tends to be genetically homogeneous but each chicken breed may not. Only when we examine commercially pure lines of Broilers and Layers, significant reduction in genetic variation is observed [44].

To examine this feature in more detail, we applied the MCMC method to two breeds; Koshamo and Ukokkei with an extremely short divergence time. The estimated θ is 0.05% for Koshamo and 0.16% for Ukokkei with τ = 0.005% and ancestral θ = 0.68%. The extent of polymorphism significantly differs from one breed to another, but most of the genetic variation can be attributed to a common ancestral population, with only a minor proportion associated with introgression. More interestingly, although the small τ value indicates 29,000 years, the two ancestral lineages leading to Koshamo and Ukokkei appear to have been distinct well before their domestication. Many distinct lineages of the ancestor may have been involved in chicken domestication, but they are not represented in the extant populations of junglefowls. If this is the case, we would not expect the MCMC estimates of species divergence times to coincide with the actual time of chicken domestication.

### Conclusion

In conclusion, domestic chickens are closely related to red junglefowl, although genetic contribution from other junglefowls remains to be answered. The ancestral population size for domestic chickens is large and each breed still maintains a substantial portion of ancestral polymorphisms at the genomic level. Despite this, domestic chickens have undergone substantial changes in morphology and physiology. In this paper, we have demonstrated that past artificial selection does not substantially reduce the genetic variation of the domestic chicken genome. If this is the case, it is inevitable to conclude that the number of genes involved in the domestication process is likely to be small.

## Supporting Information

Figure S1 Examples of individual trees. Each tree is constructed from randomly concatenated sequences (see text). Scale of each tree is indicated at the bottom of each tree. Red rectangle indicates RedDB, and green rectangle means either GJF303 or GJF304, which is not in a green junglefowl cluster. Brackets at the right side indicate a cluster of C (chickens), R (red junglefowl), and G (green junglefowl), respectively.
(0.33 MB PDF)

Figure S2 Effect of introgressed sequences from chickens to GJF on the nucleotide diversity (pai) of GJF. Pai values at each intron with and without GJF303/304 were located on both sides of the graph. Among 30 introns, intron 13 and 17 were only the exception where removal of introgressed sequences did not reduce the pai value.
(0.06 MB PDF)

Table S1 Haplotypes (numbers) of each individual.
(0.34 MB PDF)

Table S2 Two sets of scale and shape parameters (a, b) of the gamma distribution in MCMC analyses and prior distribution (%).
(0.03 MB PDF)

Table S3 The average distances of 26 concatenated intron sequences.
(0.05 MB PDF)

Table S4 Per-site nucleotide divergence (d) and standard deviation (s.d.) among chickens, RJF and GJF.
(0.33 MB PDF)

Table S5 Hardy-Weinberg equilibrium test in chicken breeds.
(0.44 MB PDF)
