# Supplementary material for: The Origin and Genetic Variation of Domestic Chickens with Special Reference to Junglefowls Gallus g. gallus and G. varius
Source: PLoS One. 2010 May 19;5(5):e10639. doi: 10.1371/journal.pone.0010639 (PMC2873279; doi:10.1371/journal.pone.0010639)
Supplement: Figure S1 — Examples of individual trees. Each tree is constructed from randomly concatenated sequences (see text). Scale of each tree is indicated at the bottom of each tree. Red rectangle indicates RedDB, and green rectangle means either GJF303 or GJF304, which is not in a green junglefowl cluster. Brackets at the right side indicate a cluster of C (chickens), R (red junglefowl), and G (green junglefowl), respectively. (0.33 MB PDF) [file pone.0010639.s001.pdf]

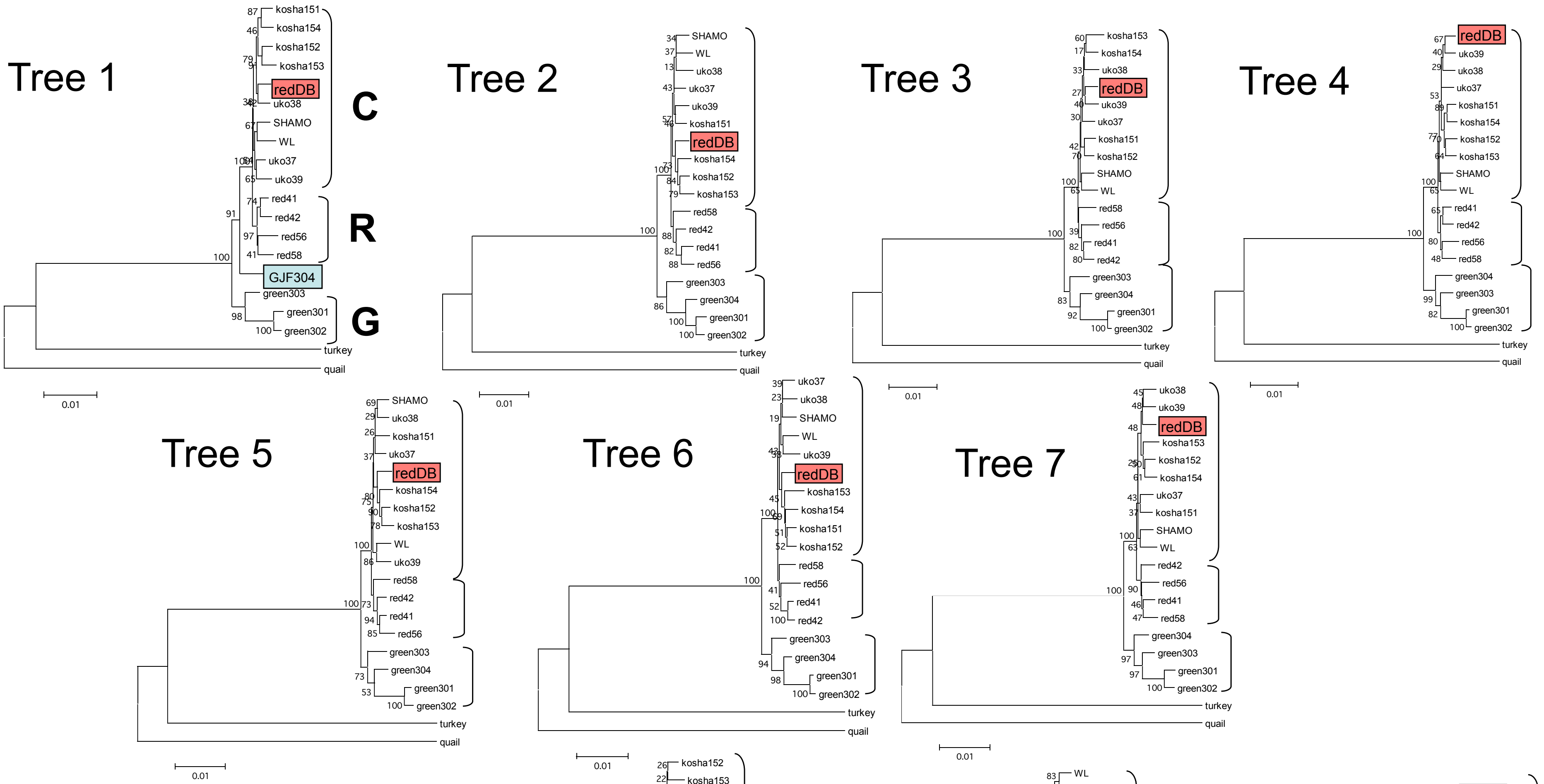

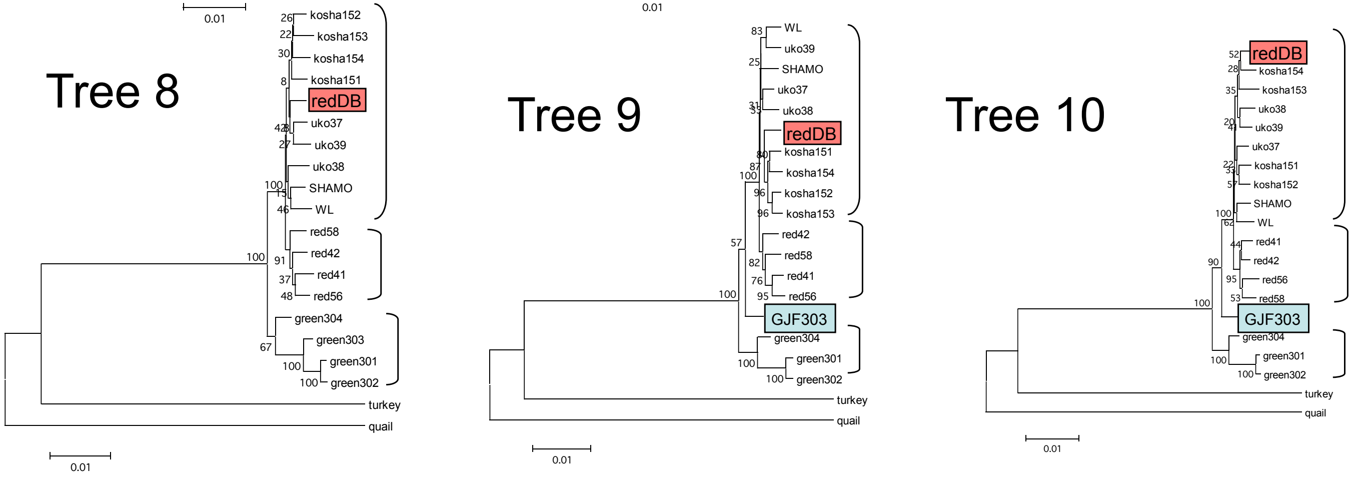

Figure S1. Examples of *individual trees*.
Each tree is constructed from randomly concatenated sequences (see text). Scale of each tree is indicated at the bottom of each tree. Red rectangle indicates “redDB,” and green rectangle means either “GJF 303” or “304”, which is not in a green junglefowl cluster. Brackets at the right side indicate a cluster of C (chickens), R(red junglefowls), and G(green junglefowls), respectively
