# Supplementary material for: The Origin and Genetic Variation of Domestic Chickens with Special Reference to Junglefowls Gallus g. gallus and G. varius
Source: PLoS One. 2010 May 19;5(5):e10639. doi: 10.1371/journal.pone.0010639 (PMC2873279; doi:10.1371/journal.pone.0010639)
Supplement: Figure S2 — Effect of introgressed sequences from chickens to GJF on the nucleotide diversity (pai) of GJF. Pai values at each intron with and without GJF303/304 were located on both sides of the graph. Among 30 introns, intron 13 and 17 were only the exception where removal of introgressed sequences did not reduce the pai value. (0.06 MB PDF) [file pone.0010639.s002.pdf]

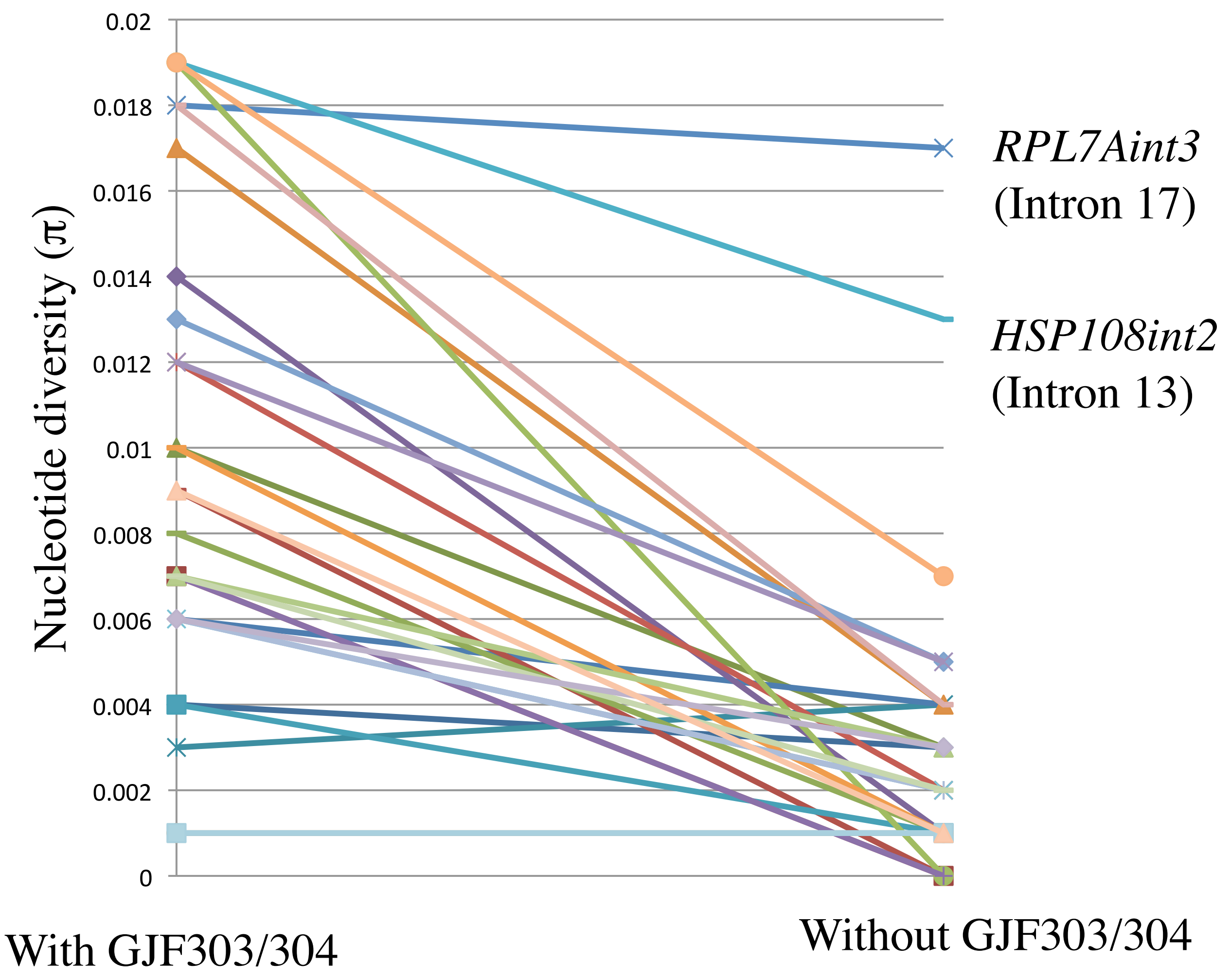

Figure S2. Effect of introgressed sequences from chickens to GJFs on the nucleotide diversity (π) of GJFs.

π values at each intron with and without GJF303/304 were located on both sides of the graph. Among 30 introns, intron 13 and 17 were only the exception where removal of introgressed sequences did not reduce the π value.
