# Supplementary material for: The Origin and Genetic Variation of Domestic Chickens with Special Reference to Junglefowls Gallus g. gallus and G. varius
Source: PLoS One. 2010 May 19;5(5):e10639. doi: 10.1371/journal.pone.0010639 (PMC2873279; doi:10.1371/journal.pone.0010639)
Supplement: Table S1 — Haplotypes (numbers) of each individual. (0.34 MB PDF) [file pone.0010639.s003.pdf]

Table S1. Haplotypes (numbers) of each individual.

	individuals																			
	SHAMO[a]	WL[a]	RedDB[a]	UKO37	UKO38	UKO39	UKO40	KOSHA151	KOSHA152	KOSHA153	KOSHA154	RJF41	RJF45	RJF56	RJF58	GJF301	GJF302	GJF303	GJF304
intron 1	1	3	–[c]	4/4	5/6	4/4	4/4	7/7	6/6	6/8	6/6	4/9	2/10	9/10	10/10	6/11	12/12	4/6	5/6
intron 2	1	2	–	6/7	6/8	3/5a	–	5a/5b	5a/6	5a/6	9/9	3/10	10/10	3/4	4/4	11/11	11/11	6/11	6/12
intron 3	1	2	3	4/5	6/7	3/8	3/4	3/3	3/4	3/4	3/3	9/10	10/10	7/9	7/7	11/12	11/13	4/14	4/11
intron 4	1	2	3	–	–	–	–	1/3	3/3	3/3	1/1	3/4	3/5	3/6	–	–	–	–	–
intron 5	1	1	1	1/1	1/2	1/1	2/2	1/1	1/1	1/1	1/1	2/2	2/2	2/2	2/2	3/4	3/3	2/3	2/2
intron 6	1	2	3	2/4	4/5	1/1	5/6	4/4	2/2	1/2	1/7	2/2	2/5	5/8	2/9	10/10	10/11	2/10	2/10
intron 7	1	1	–	2/3	1/1	2/2	4/4	2/2	1/2	5/6	1/1	1/6	7/8	8/9	6/8	10/10	11/11	10/13	5/12
intron 8	1	1	2	3/3	4/4	2/2	-	2/2	1/1	5/6	4/4	4/7	7/8	1/7	7/9	10/10	10/10	5/10	11/11
intron 9	1	2	3	4/5	1/6	1/4	-	1/1	4/4	1/1	3/3	7/8	4/9	7/9	4/9	10/11	10/10	3/10	5/10
intron 10	1a[b]	2	2	3/4	5/1b	1b/6	7/7	8/8	6/6	1b/9	1b/1b	10/10	1b/11	10/12	10/13	14/15	15/16	1b/17	18/19
intron 11	1	2	3	4/4	4/5	6/6	5/7	8/8	9/9	10/11	12/12	13/13	5/13	5/14	10/15	16/17	17/17	17/18	17/17
intron 12	1	2	3	2/2	4/5	2/2	5/6	7/7	7/7	2/3	2/2	2/3	3/6	6/6	6/6	8/9	8/8	3/8	10/10
intron 13	1	2	2	2/2	3/4	2/4	-	5/5	4/4	4/6	7/7	5/8	5/9	10/10	1/1	11/12	13/14	1/12	5/12
intron 14	1	2	3a	4/5	2/6	4/4	3a/7	2/3a	3a/3b	3b/8	3a/3b	2/3a	2/3a	9/9	3a/3a	10/10	11/11	5/12	5/11
intron 15	1	2	1	3/3	4/4	4/4	-	4/4	5/6	6/6	3/4	7/8	9/9	8/8	8/8	10/10	10/10	6/6	3/11
intron 16	1	2	2	2/3	2/4	2/2	1/1	3/3	3/3	2/5	3/6	2/7	7/7	2/7	8/8	9/9	9/9	1/10	2/11
intron 17	1	2	3	4/4	4/5	6/6	4/7	4/4	4/4	4/4	8/9	10/10	11/11	10/12	11/13	14/15	16/17	8/18	15/19
intron 18	1	2	3	1a/1b	4/4	5/5	3/6	3/3	3/3	7/7	8/8	1a/1a	7/7	1a/1a	1a/9	-	-	-	-
intron 19	1	2a	3	1/1	3/4	1/2b	4/4	1/1	1/5	5/5	1/1	1/6	6/7	7/8	8/8	9/10	9/9	1/11	4/9
intron 20	1	2	3	4/4	5/5	2/2	6/6	7/7	8/9	7/9	7/7	10/10	10/11a	2/10	11b/11b	12/12	13/14	9/15	2/12
intron 21	1	2	3	4/5	5/6	5/5	-	7/8	7/7	9/10	8/9	11/11	11/11	11/11	12/12	13/14	13/15	6/6	16/16
intron 22	1	2	2	3/3	4/4	5/5	-	3/3	2/2	2/2	6/6	4/7	4/4	4/8	4/8	9/9	10/10	3/10	3/10
intron 23	1	2	3	4/4	3/5	4/5	6/6	4/4	3/3	3/3	6/6	5/6	5/7	5/7	5/7	8/9	8/8	8/8	4/8
intron 24	1	2	2	3/4	5/5	5/5	6/6	4/4	2/2	2/2	6/6	1/5	5/5	5/5	5/5	7/8	8/8	1/8	4/8
intron 25	1	2	3	1/4	1/5	6/7	8/8	9/10	10/10	10/10	11/11	12/13	14/14	14/15	10/16	17/17	18/18	19/20	21/22
intron 26	1	2	3	4/4	5/6	7/7	-	5/8	5/9	10/11	11/12	13/13	13/14	4/15	13/14	16/17	18/19	4/20	21/22
intron 27	1	1	1	1/1	1/1	2/2	1/1	1/1	3/3	1/1	3/3	1/1	1/4	1/1	1/5	6/7	6/7	6/8	4/6
intron 28	1	2	3	4/4	1/3	3/5	3/3	3/6	3/6	3/3	3/3	4/7	4/4	4/4	7/7	8/9	10/11	10/12	8/12
intron 29	1	1	1	1/1	1	1	1	2/3	2	2/2	1	1	1/1	1/1	1/1	4/5	5/6	7	7
intron 30	1	2	3	3/3	1/3	3/3	1/1	1/1	1/1	4/4	1/1	1/5	1/3	1/1	6/7	8/8	9/9	3/8	9/10

[a] A single sequence at each intron was obtained from SHAMO, WL, and RedDB.
[b] A haplotype number that is followed by a letter indicates that the haplotype differed from another haplotype (as indicated by the same number followed by a different letter) because of indels.
[c] A dash indicates that sequences were not available.
