# Supplementary material for: The Origin and Genetic Variation of Domestic Chickens with Special Reference to Junglefowls Gallus g. gallus and G. varius
Source: PLoS One. 2010 May 19;5(5):e10639. doi: 10.1371/journal.pone.0010639 (PMC2873279; doi:10.1371/journal.pone.0010639)
Supplement: Table S2 — Two sets of scale and shape parameters (a, b) of the gamma distribution in MCMC analyses and prior distribution (%). (0.03 MB PDF) [file pone.0010639.s004.pdf]

Table S2. Two sets of scale and shape parameters (a, b) of the gamma distribution in MCMC analyses and prior distribution (%).

Parameters	Set 1[a]		Set 2[b]	
	(a, b)	Prior [95% interval]	(a, b)	Prior [95% interval]
θ_C	4, 650	0.62 [0.17, 1.35]	19, 7200	0.26 [0.16, 0.40]
θ_R	3, 580	0.52 [0.11, 1.25]	13, 12000	0.11 [0.06, 0.18]
θ_G	3, 320	0.94 [0.19, 2.26]	32, 9400	0.34 [0.23, 0.47]
θ_{CR}	4, 600	0.67 [0.18, 1.46]	26, 3600	0.72 [0.47, 1.03]
θ_{RCG}	4, 600	0.67 [0.18, 1.46]	114, 7100	1.61 [1.32, 1.91]
τ_{CR}	1,50000	0.002 [0, 0.007]	1, 11000	0.01 [0, 0.034]
τ_{RCG}	16, 2500	0.64 [0.37, 0.99]	113, 18000	0.63 [0.52, 0.75]

[a] Parameters were calculated based on observed data.
[b] Parameters were based on posteriors using parameters Set 1(see Table 4).
