# Supplementary material for: The Origin and Genetic Variation of Domestic Chickens with Special Reference to Junglefowls Gallus g. gallus and G. varius
Source: PLoS One. 2010 May 19;5(5):e10639. doi: 10.1371/journal.pone.0010639 (PMC2873279; doi:10.1371/journal.pone.0010639)
Supplement: Table S3 — The average distances of 26 concatenated intron sequences. (0.05 MB PDF) [file pone.0010639.s005.pdf]

Table S3. The average distances of 26 concatenated intron sequences.

	SHAMO	WL	UKO37	UKO38	UKO39	KOSHA 151	KOSHA 152	KOSHA 153	KOSHA 154	RedDB	RJF41	RJF42	RJF56	RJF58	GJF301	GJF302	GJF303	GJF304	TURKEY
WL	0.006																		
UKO37	0.006	0.006																	
UKO38	0.006	0.007	0.006																
UKO39	0.007	0.006	0.005	0.007															
KOSHA151	0.006	0.008	0.006	0.007	0.007														
KOSHA152	0.006	0.007	0.006	0.007	0.007	0.005													
KOSHA153	0.007	0.009	0.008	0.007	0.008	0.006	0.005												
KOSHA154	0.007	0.009	0.006	0.007	0.007	0.005	0.006	0.006											
RedDB	0.008	0.007	0.006	0.007	0.006	0.006	0.007	0.006	0.006										
RJF41	0.006	0.007	0.007	0.008	0.008	0.008	0.008	0.008	0.007	0.007									
RJF42	0.007	0.007	0.006	0.007	0.008	0.007	0.007	0.008	0.007	0.007	0.004								
RJF56	0.008	0.008	0.008	0.009	0.009	0.008	0.008	0.008	0.008	0.008	0.005	0.006							
RJF58	0.007	0.008	0.007	0.008	0.008	0.007	0.007	0.009	0.007	0.007	0.006	0.006	0.007						
GJF301	0.018	0.017	0.017	0.017	0.017	0.016	0.017	0.017	0.017	0.017	0.017	0.017	0.017	0.017					
GJF302	0.018	0.017	0.018	0.018	0.018	0.017	0.017	0.017	0.017	0.017	0.017	0.017	0.016	0.017	0.003				
GJF303	0.011	0.012	0.011	0.012	0.012	0.011	0.012	0.013	0.012	0.011	0.011	0.011	0.012	0.012	0.010	0.010			
GJF304	0.012	0.013	0.012	0.012	0.013	0.012	0.013	0.013	0.013	0.013	0.013	0.013	0.013	0.013	0.009	0.009	0.010		
TURKEY	0.099	0.099	0.099	0.099	0.099	0.098	0.099	0.099	0.099	0.099	0.099	0.099	0.100	0.099	0.100	0.100	0.099	0.099	
QUAIL	0.111	0.111	0.111	0.110	0.111	0.110	0.110	0.110	0.111	0.110	0.110	0.110	0.110	0.110	0.110	0.110	0.110	0.110	0.121

The average p-distances were calculated over 1,000 sampling of concatenated intron sequences.

An individual sequence is a concatenated sequence that was randomly selected from the diploid sequence for 26 introns.

Four introns were not used because sequences are not available for a few individual samples.

Only a single sequence was available for all introns from SHAMO, WL and RedDB.
