# Supplementary material for: The Origin and Genetic Variation of Domestic Chickens with Special Reference to Junglefowls Gallus g. gallus and G. varius
Source: PLoS One. 2010 May 19;5(5):e10639. doi: 10.1371/journal.pone.0010639 (PMC2873279; doi:10.1371/journal.pone.0010639)
Supplement: Table S4 — Per-site nucleotide divergence (d) and standard deviation (s.d.) among chickens, RJF and GJF. (0.33 MB PDF) [file pone.0010639.s006.pdf]

Table S4. Per-site nucleotide divergence (*d*) and standard deviation (s.d.) among chickens, RJF and GJF.

	Per-site nucleotide divergence (*d*)					
Intron	Chickens vs. RJF	s.d.	Chickens vs. GJF	s.d.	RJF vs. GJF	s.d.
1	0.008	0.004	0.005	0.004	0.007	0.003
2	0.01	0.004	0.013	0.006	0.015	0.004
3	0.015	0.005	0.015	0.008	0.028	0.009
4	0.003	0.002	N.A.[a]	N.A.	N.A.	N.A.
5	0.003	0.006	0.005	0.006	0.002	0.002
6	0.003	0.002	0.003	0.002	0.003	0.001
7	0.004	0.003	0.009	0.003	0.009	0.003
8	0.011	0.006	0.026	0.004	0.022	0.005
9	0.008	0.004	0.016	0.006	0.017	0.007
10	0.017	0.003	0.025	0.008	0.029	0.008
11	0.007	0.003	0.012	0.004	0.011	0.002
12	0.01	0.005	0.017	0.007	0.02	0.007
13	0.017	0.01	0.024	0.01	0.022	0.009
14	0.006	0.004	0.015	0.006	0.014	0.003
15	0.006	0.004	0.022	0.014	0.022	0.014
16	0.006	0.003	0.012	0.005	0.013	0.005
17	0.015	0.005	0.026	0.009	0.023	0.007
18	0.005	0.003	N.A.	N.A.	N.A.	N.A.
19	0.008	0.005	0.015	0.006	0.014	0.005
20	0.007	0.004	0.018	0.007	0.017	0.008
21	0.005	0.001	0.01	0.005	0.011	0.003
22	0.01	0.004	0.018	0.008	0.019	0.006
23	0.011	0.006	0.02	0.008	0.018	0.005
24	0.004	0.003	0.031	0.015	0.029	0.014
25	0.005	0.002	0.012	0.004	0.012	0.004
26	0.012	0.005	0.03	0.011	0.031	0.011
27	0.002	0.004	0.009	0.003	0.009	0.003
28	0.012	0.006	0.018	0.007	0.011	0.004
29	0.001	0.001	0.004	0.001	0.004	0.001
30	0.004	0.003	0.015	0.005	0.015	0.005

[a] N.A. indicates “not available”.
