# Supplementary material for: The Origin and Genetic Variation of Domestic Chickens with Special Reference to Junglefowls Gallus g. gallus and G. varius
Source: PLoS One. 2010 May 19;5(5):e10639. doi: 10.1371/journal.pone.0010639 (PMC2873279; doi:10.1371/journal.pone.0010639)
Supplement: Table S5 — Hardy-Weinberg equilibrium test in chicken breeds. (0.44 MB PDF) [file pone.0010639.s007.pdf]

Table S5. Hardy-Weinberg equilibrium test in chicken breeds.

Intron[a]	Genotype of individuals								Frequency of haplotypes	Expected homozygosity	Expected F[b]	Observed F[c]	χ^2 [d]
	UKO37	UKO38	UKO39	UKO40	KOSHA151	KOSHA152	KOSHA153	KOSHA154					
1	4/4	5/6	4/4	4/4	7/7	6/6	6/8	6/6	4(6/16), 5 (1/16), 6 (6/16), 7(2/16), 8(1/16)	0.30	2.4	6	5.40
2	6/7	6/8	3/5a	–	5a/5b	5a/6	5a/6	9/9	3(1/14), 5(5/14), 6(4/14), 7(1/14), 8(1/14), 9 (2/14)	0.24	1.7	2	0.05
3	4/5	6/7	3/8	3/4	3/3	3/4	3/4	3/3	3 (8/16), 4 (4/16), 5 (1/16), 6 (1/16), 7 (1/16), 8(1/16)	0.33	2.6	2	0.14
4	–	–	–	–	1/3	3/3	3/3	1/1	1(3/8), 3(5/8)	0.53	2.1	3	0.39
5	1/1	1/2	1/1	2/2	1/1	1/1	1/1	1/1	1(13/16), 2(3/16)	0.7	5.7	7	0.30
6	2/4	4/5	1/1	5/6	4/4	2/2	1/2	1/7	1(4/16),2(4/16),4(4/16),5(2/16),6 (1/16),7(1/16)	0.21	1.7	3	0.99
7	2/3	1/1	2/2	4/4	2/2	1/2	5/6	1/1	1(5/16), 2(6/16), 3(1/16), 4(2/16), 5(1/16), 6(1/16)	0.27	2.1	5	4.00
8	3/3	4/4	2/2	-	2/2	1/1	5/6	4/4	1(2/14), 2(4/14), 3(2/14), 4(4/14), 5(1/14), 6(1/14)	0.21	1.5	6	13.50
9	4/5	1/6	1/4	-	1/1	4/4	1/1	3/3	1(6/14), 3(2/14), 4(4/14), 5(1/14), 6(1/14)	0.3	2.1	4	1.72
10	3/4	5/1b	1b/6	7/7	8/8	6/6	1b/9	1b/1b	1(5/16), 3(1/16), 4(1/16), 5(1/16), 6(3/16), 7(2/16), 8(2/16), 9(1/16)	0.18	1.4	4	4.83
11	4/4	4/5	6/6	5/7	8/8	9/9	10/11	12/12	4(3/16), 5(2/16), 6(2/16), 7(1/16), 8(2/16), 9(2/16),10(1/16), 11(1/16), 12 (2/16)	0.13	1	5	16.00
12	2/2	4/5	2/2	5/6	7/7	7/7	2/3	2/2	2(7/16), 3(1/16), 4(1/16), 5(2/16), 6(1/16), 7(4/16)	0.28	2.3	5	3.17
13	2/2	3/4	2/4	-	5/5	4/4	4/6	7/7	2(3/14), 3(1/14), 4(5/14), 5(2/14), 6(1/14), 7(2/14)	0.22	1.6	4	3.60
14	4/5	2/6	4/4	3a/7	2/3a	3a/3b	3b/8	3a/3b	2(2/16), 3(7/16), 4(3/16), 5(1/16), 6(1/16), 7(1/16), 8(1/16)	0.26	2.1	3	0.39
15	3/3	4/4	4/4	-	4/4	5/6	6/6	3/4	3(3/14), 4(7/14), 5(1/14), 6(3/14)	0.34	2.4	5	2.82
16	2/3	2/4	2/2	1/1	3/3	3/3	2/5	3/6	1(2/16), 2(5/16), 3(6/16), 4(1/16), 5(1/16), 6(1/16)	0.27	2.1	4	1.72
17	4/4	4/5	6/6	4/7	4/4	4/4	4/4	8/9	4(10/16), 5(1/16), 6(2/16), 7(1/16), 8(1/16), 9(1/16)	0.43	3.4	5	0.75
18	1a/1b	4/4	5/5	3/6	3/3	3/3	7/7	8/8	1(2/16), 3(5/16), 4(2/16), 5(2/16), 6(1/16), 7(2/16), 8(2/16)	0.18	1.4	7	22.40
19	1/1	3/4	1/2b	4/4	1/1	1/5	5/5	1/1	1(8/16), 2(1/16), 3(1/16), 4(3/16), 5(3/16)	0.33	2.6	5	2.22
20	4/4	3/4	2/2	6/6	7/7	8/9	7/9	7/7	2(2/16), 4(2/16), 5(2/16), 6(2/16), 7(5/16), 8(1/16), 9(1/16)	0.17	1.3	6	16.99
21	4/5	5/6	5/5	-	7/8	7/7	9/10	8/9	4(1/14), 5(4/14), 6(1/14), 7(3/14), 8(2/14), 9(2/14), 10(1/14)	0.18	1.3	2	0.38
22	3/3	4/4	5/5	-	3/3	2/2	2/2	6/6	2(4/14), 3(4/14), 4(2/14), 5(2/14), 6(2/14)	0.22	1.6	7	18.23
23	4/4	3/5	4/5	4/6	4/4	3/3	3/3	6/6	3(5/16), 4(6/16), 5(2/16), 6(3/16)	0.29	2.3	5	3.17
24	3/4	5/5	5/5	4/6	4/4	2/2	2/2	6/6	2(4/16), 3(1/16), 4(4/16), 5(4/16), 6(3/16)	0.23	1.8	6	9.80
25	1/4	1/5	6/7	8/8	9/10	10/10	10/10	11/11	1(2/16), 4(1/16), 5(1/16), 6(1/16), 7(1/16), 8(2/16), 9(1/16), 10(5/16), 11(2/16)	0.16	1.3	4	5.61
26	4/4	5/6	7/7	-	5/8	5/9	10/11	11/12	4(2/14), 5(3/14), 6(1/14), 7(2/14), 8(1/14), 9(1/14), 10(1/14), 11(2/14), 12(1/14)	0.13	0.9	2	1.34
27	1/1	1/1	2/2	1/1	1/1	3/3	1/1	3/3	1(10/16), 2(4/16), 3(4/16)	0.52	4.1	8	3.71
28	4/4	1/3	3/5	3/3	3/6	3/6	3/3	3/3	1(1/16), 3(10/16), 4(2/16), 5(1/16), 6(2/16)	0.43	3.4	4	0.11
30	3/3	3/3	3/3	1/1	1/1	1/1	4/4	1/1	1(9/16), 3(5/16), 4(2/16)	0.43	3.4	7	3.81
# of loci examined	29	28	28	22	30	29	30	29					
# of homozygous introns	17	8	19	11	22	20	15	22					

[a] Intron29 was excluded because it is on Z chromosome.
[b] The expected number of homozygotes. Total number of expected homozygotes was 63.6.
[c] The observed number of homozygotes. Total number of observed homozygotes was 136.
[d] The chi-square value was calculated for each intron. The sum of chi-square values was 147.52. This result was highly significant ($P < 0.001$) with d.f. = 28.
